# Tetraspanin18 regulates angiogenesis through VEGFR2 and Notch pathways

**DOI:** 10.1242/bio.050096

**Published:** 2021-02-25

**Authors:** Grace X. Li, Shaobing Zhang, Ren Liu, Bani Singh, Sukhmani Singh, David I. Quinn, Gage Crump, Parkash S. Gill

**Affiliations:** 1 Department of Medicine, Keck School of Medicine of the University of Southern California, Los Angeles, California; 2 Department of Medicine, USC Norris Comprehensive Cancer Center, Keck School of Medicine, University of Southern California, Los Angeles, CA, USA; 3 Department of Neurobiology, Keck School of Medicine of the University of Southern California, Los Angeles, CA, USA

**Keywords:** Tspan18, VEGFR2, Notch, Angiogenesis, Zebrafish

## Abstract

The VEGF pathway is critically required for vasculogenesis, the formation of the primary vascular network. It is also required for angiogenesis resulting in sprouting and pruning of vessels to generate mature arborizing structures. The Notch pathway is essential for arterial–venous specification and the maturation of nascent vessels. We have determined that Tspan18, a member of the Tetraspanin family, is expressed in developing vessels but not in mature vasculature in zebrafish and mouse wound healing. Moreover, reduction at Tspan18 level resulted in aberrant vascular patterning, impaired vessel stability and defective arterial–venous specification. Tspan18 deficiency reduced VEGF, VEGFR2, Notch3 and EphrinB2, and increased EphB4, VEGFR3, Semaphorin3, Neuropilin and PlexinD1 expression. Furthermore, vascular defects of Tspan18 deficiency could be rescued by ectopic expression of VEGFR2 and Notch, but not by knockdown of Semaphorin or Plexin. Functional studies showed that knockdown of Tspan18 led to reduced endothelial cell migration, invasion and tube formation. Tspan18 has dynamic expression, regulates vascular development and maturation in the embryo with re-expression in adult life in wound healing.

## Abstract (precis)

**Summary:** Tspan18 is a transmembrane protein with highly restricted expression in endothelial cells and a critical regulator of VEGF and Notch pathways. It regulates artery–vein specification, vessel patterning and vessel stability.

## INTRODUCTION

The development of the embryonic vasculature begins *de novo* from mesodermal precursors (Carmeliet, 2004) in the form of a primary capillary plexus. Next, angiogenesis, which involves the sprouting, pruning and remodeling of capillaries, generates an arborizing mature vascular system. During this process, endothelial cells (EC) acquire an arterial phenotype determined by the expression of Notch1, Notch4, Notch-ligand Delta-like 4 (Dll4) and EphrinB2 (Lawson and Weinstein, 2002a), and a venous phenotype by the expression of EphB4 and vascular endothelial growth factor receptor 3 (VEGFR3) (Swift and Weinstein, 2009). Arterial–venous identity is established before blood flow is established (Carmeliet et al., 1996).

Vascular endothelial growth factor (VEGF) signaling is crucial for both vasculogenesis and angiogenesis (Ferrara et al., 1996; Ferrara and Davis-Smyth, 1997). VEGF binds to three distinct type III receptor tyrosine kinases: VEGFR1/Flt-1, VEGFR2/Flk-1/Kdr and VEGFR3/Flt4. VEGF/VEGFR2 signaling is required for the formation of the primary vascular network and the subsequent sprouting and pruning of vessels during angiogenesis (Alitalo and Carmeliet, 2002; Shibuya and Claesson-Welsh, 2006; Olsson et al., 2006; Roca and Adams, 2007). VEGF also regulates the Notch pathway and thus arterial–venous specification (Phng and Gerhardt, 2009; Kageyama et al., 2007; Siekmann and Lawson, 2007). Loss of Notch signaling leads to the loss of arterial markers, gain of venous markers and an increase in vascular sprouting (Tarrant et al., 2003).

We report the discovery of a novel key regulator of angiogenesis, tetraspanin 18 (Tspan18). Tspan18 is one of 33 members of the tetraspanin family. Tetraspanins generate multimolecular complexes resulting in microdomains distinct from lipid rafts. Although tetraspanins typically lack ligands, the tetraspanin microdomains function as signaling complexes in the plasma membrane by interacting with many membrane proteins (Hemler, 2003; Wright et al., 2004; Lawson and Weinstein, 2002b). We demonstrate that Tspan18 is expressed in the blood vessels, and is required for proper angiogenesis including vessel patterning, vessel stability and arterial–venous specification. Tspan18 signaling also regulates VEGF and Notch pathways and wound healing.

## RESULTS

**Fig. 1.**
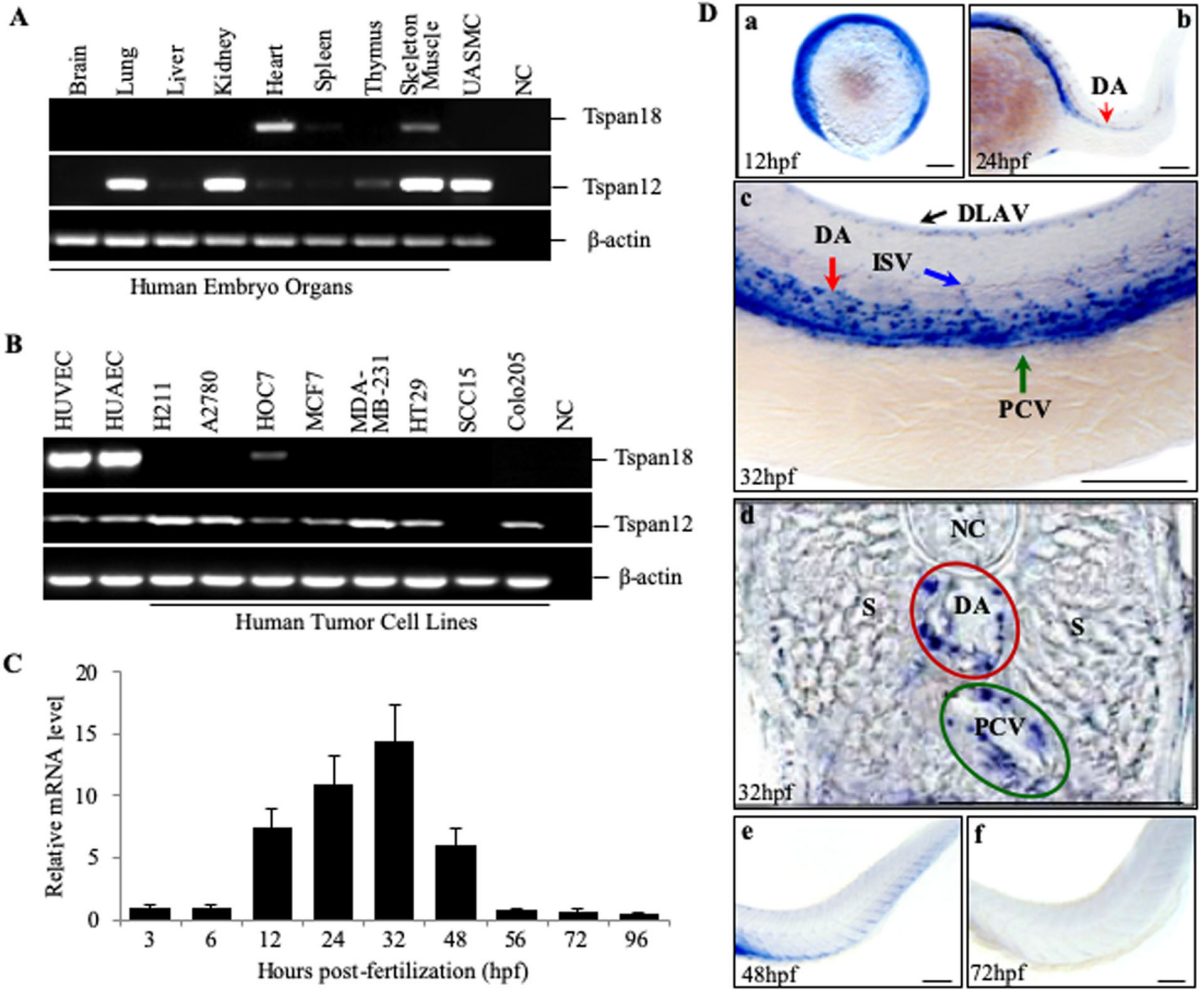
**Tspan18 expression in the cardiovascular system.** The expression of Tspan18 and its paralogue Tspan12 was analyzed by RT-PCR in (A) human fetal tissues and (B) primary cultured endothelial cells and tumor cell lines. UASMC, umbilical artery smooth muscle cell; HUVEC, human umbilical vein endothelial cell; HUAEC, human umbilical artery endothelial cell; NC, negative control with no cDNA. (C) Quantitative RT-PCR analysis of Tspan18 expression at different times in zebrafish development (whole embryo). Expression was normalized to β–actin. (D) Whole-mount ISH of Tspan18 expression in the developing embryos. D is the cross section of C. DA, dorsal aorta; PCV, posterior cardinal vein; DLAV, dorsal longitudinal anastomotic vessels; ISV, intersegmental vessel; S, somites; NC, Notochord. All larvae lateral view unless indicated. Anterior is to the left. Scale bars:100 μm. All experiments (A–D) were repeated at least three times and similar results were obtained.

We discovered Tspan18 during the screen for genes expressed specifically in the endothelium. Tspan18 showed preferential expression in endothelial cells but not adult normal tissues (data not shown). Tspan18 is highly conserved in vertebrates (Fig. S1). Human embryonic tissue analysis showed expression in the heart, low-level expression in the skeletal muscle (Fig. 1A) and high expression in primary endothelial cells (Fig. 1B). No expression was observed in the lungs, kidney, liver, spleen, colon, brain and most of the human cancer cell lines screened. In comparison, tetraspanin 12 (Tspan12, a paralogue of Tspan18), recently shown to be expressed in retinal vessels (Junge et al., 2009), was widely expressed in embryonic tissues as well as human tumor cell lines (Fig. 1A,B).

We first studied Tspan18 function in zebrafish because of the rapid development of their vascular systems, the ability to monitor real-time vascular development, and the similarity in the development of their vascular system to mammals’. We used both quantitative RT-PCR and whole mount *in situ* hybridization (ISH) to assess the expression of Tspan18. The expression first appeared at early somitogenesis stage, 12 h post fertilization (hpf), in yolk marginal zone (Fig. 1C and Da), and then specifically in the dorsal aorta (DA) and in yolk vessels at 24 hpf (Fig. 1Db). At 32 hpf, its expression was observed in the DA, posterior cardinal vein (PCV), dorsal longitudinal anastomotic vessels (DLAV) and intersegmental vessel (ISV) (Fig. 1Dc and d). Tspan18 expression in these vessels was maintained through 32 hpf and then reduced from 32 to 48 hpf (Fig. 1C and De). We also observed Tspan18 expression in the central nervous system at 48 h (data not shown), as previously reported (Fairchild and Gammill, 2010). Tspan18 expression was almost undetectable from 56 hpf (Fig. 1C and Ae) to 4 days post fertilization (dpf) (Fig. 1C). The decline in Tspan18 coincides with maturation of vascular system thus suggesting a dynamic expression corresponding to vascular development. Mouse Tspan18 expression is localized to the developing heart of 10.5 dpc embryo (Fig. S2A), intersomatic vessel of 13.5 dpc embryo, adult heart, artery and vein (Fig. S2B).

**Fig. 2.**
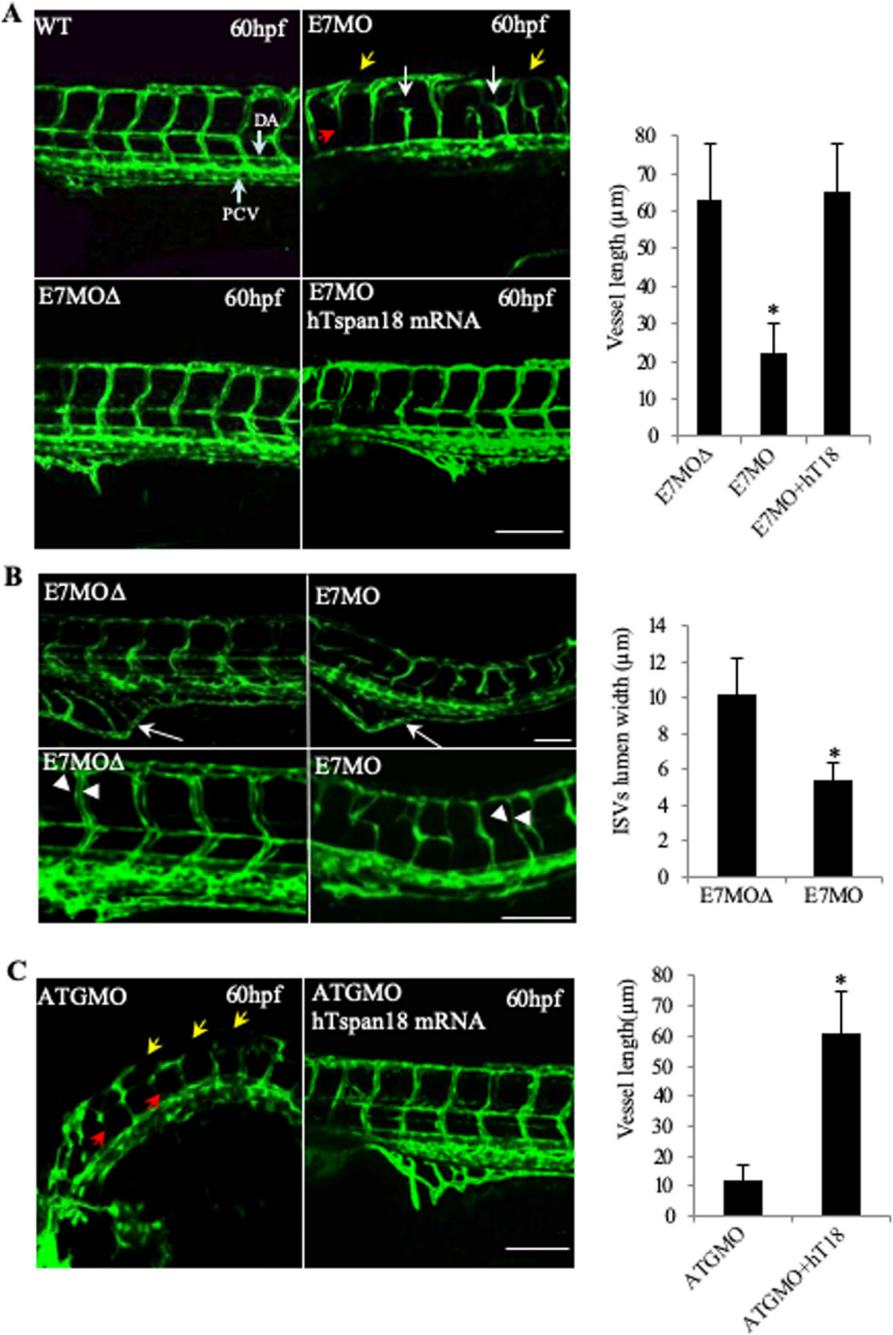
**Vascular defects in Tspan18-zebrafish morphants.** (A) Morphological and vascular defects were examined in E7MO-injected zebrafish at 60 hpf. E7MO represents the morpholino targeting Tspan18 Exon 7 splicing site. E7MOΔ is a control morpholino with five-base mismatch. WT represents fish injected with morpholino diluent only. Arrows in the picture of Tspan18-deficient fish (E7MO) point to missing ISV (red), ectopic ISV branching (white) or discontinuous DLAV (yellow). Quantification of ISV length of E7MO and E7MOΔ injected fish (*n*=80) are shown on the bottom left. Co-injection of a human sense capped Tspan18 mRNA rescued the defects caused by E7MO. (B) E7MOΔ injected embryos had reduced subintestinal vessels (upper panel, arrows) and narrow ISV lumen (lower panel, arrowheads) at 72 hpf. Quantification of ISV lumen width from each group is shown on the right (*n*=75). (C) Morphological and vascular defects were examined in ATGMO injected zebrafish at 60 hpf. ATGMO represents the morpholino targeting Tspan18 ATG start codon containing Exon 3 splicing site. Arrows are designated as in A. Co-injection of a human sense capped Tspan18 mRNA rescued the defects caused by ATGMO. Error bars represent s.d. *P* value was calculated using two-tailed Student's *t*-test. *P<0.02. All larvae lateral view. Anterior is to the left. Scale bars: 50 μm. All experiments (A–C) were repeated at least three times and similar results were obtained.

Analysis of Tspan18 function in vascular development was performed in the transgenic fish line Tg(*fli1a*:eGFP)^y1^, in which enhanced green fluorescent protein expressed under the control of the *fli1a* promoter allows monitoring of the developing vasculature (Lawson and Weinstein, 2002b). Tspan18 expression was reduced by injection of morpholino oligomers (MOs) designed to block splicing (morpholino targeting exon 7 of zebrafish Tspan18, E7MO) of the *tspan18* gene mRNA transcripts (Fig. S3A). RT-PCR confirmed that the E7MO targeted exon7 splice-donor site, blocked splicing and excluded the exon7-encoded hypervariable region from the transcript (Fig. S3B). Whereas E7MO fully blocked splicing at 24 hpf, partial recovery of full-length transcript occurred by 54 hpf, likely due to decay of MO over time (Fig. S3B). Tg(*fli1a*:eGFP)^y1^ imaging revealed multiple defects in the vasculature, including short or missing ISVs, narrow or absent lumens in the ISVs, and discontinuous DLAV (Fig. 2A,B). In normal embryos, each ISV ended dorsally in the DLAV making a T-shaped junction. Tspan18 E7MO embryos however showed ectopic branching and defective interconnections of ISVs prior to fusing with DLAV (Fig. 2A). Quantification of ISV lumens at 72 hpf showed a significantly smaller lumen width in the Tspan18 E7MO embryos compared to controls: 5.4±1.3 μm vs 10.2±2.2 μm (*P*=0.013) (Fig. 2B). Subintestinal vessel was also reduced in E7MO embryos (Fig. 2B). Axial vessels including the dorsal aorta and posterior cardinal vein however appeared normal (Fig. 2A), suggesting that Tspan18 regulates vascular development post-vasculogenesis and primarily during angiogenesis. The efficacy of this knockdown approach was confirmed using a second morpholino directed against the 5′-untranslated region (UTR) spanning the ATG start codon to inhibit translation (Fig. S3A). Both morpholinos produced a similar fish phenotype *in vivo* (Fig. 2A and C), which was not observed in embryos injected with a five-base mismatch control morpholino (Fig. 2A and C). In order to further confirm that the observed phenotype is specific to Tspan18 deficiency, E7MO was co-injected with human capped Tspan18 mRNA, which led to near complete rescue (90%±1%) of the fish phenotype (Fig. 2A, top left and bottom right) confirming the specificity of the Tspan18-deficient phenotype. Similarly, human capped Tspan18 mRNA rescued the phenotype when co-injected with morpholino-targeting ATG containing exon 3 of zebrafish Tspan18 (ATGMO) (Fig. 2C).

**Fig. 3.**
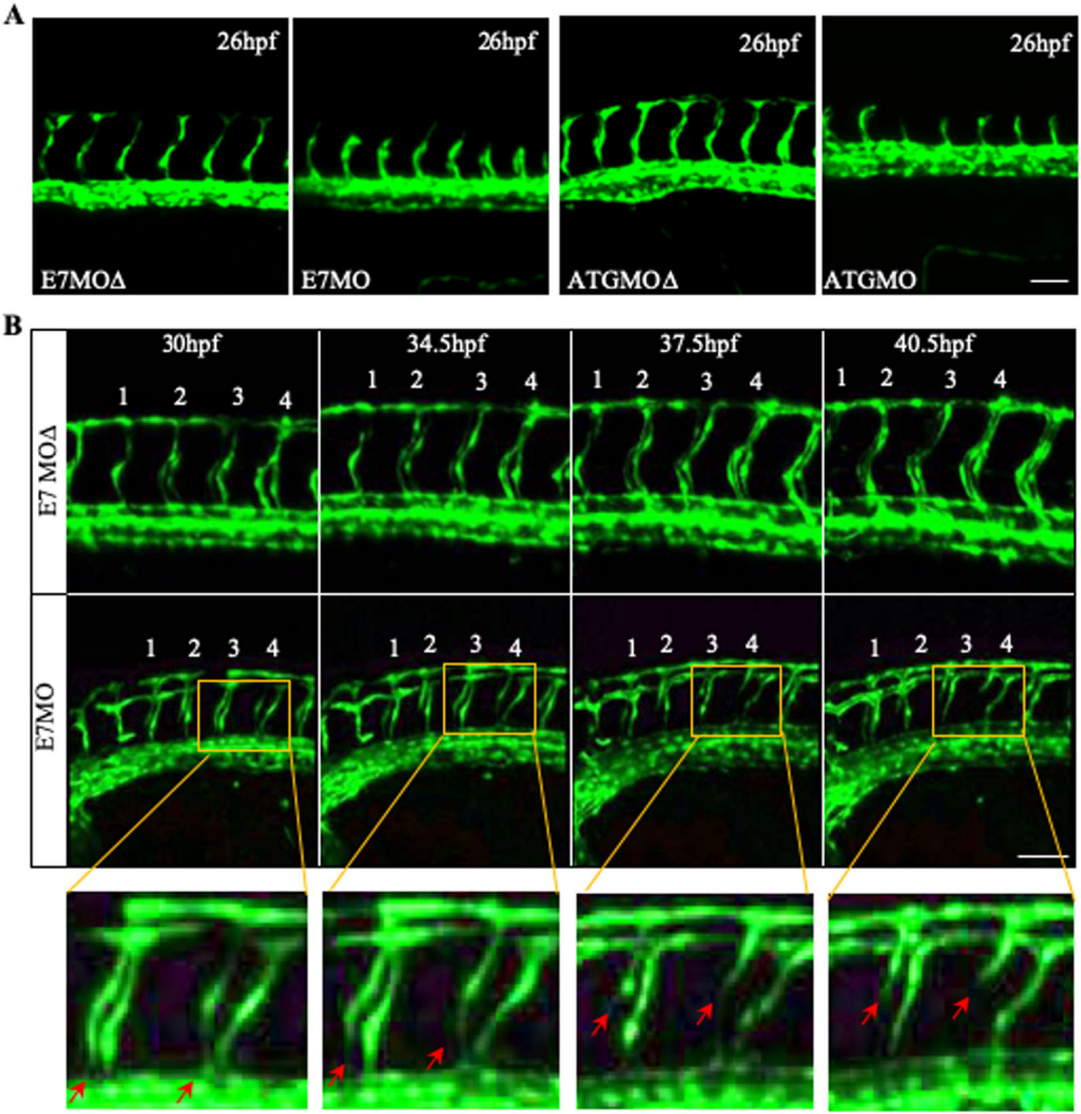
**Vessel regression in Tspan18 zebrafish morphants.** (A) Intact ISV sprouting is observed at 26 hpf in Tspan18-deficient fish. (B) Confocal time-lapse microscopy of regressing ISVs (red arrows) in Tspan18-deficient fish. Numbers 1–4 indicate the order of ISVs. Yellow rectangle: corresponding section shown at higher magnification under the bottom. All larvae lateral view. Anterior is to the left. Scale bars: 50 μm. All experiments (A–B) were repeated at least three times and similar results were obtained.

The missing ISVs in E7MO and ATGMO treated zebrafish could be caused by either impaired vessel sprouting or stability. At the early stage of ISV development, we observed no defect in the sprouting of ISVs at 26 hpf, although overall vessel development was slightly delayed (Fig. 3A). It is likely that vessel instability leads to the missing ISV phenotype. We therefore examined the fate of ISV in Tspan18 morphants by confocal time-lapse microscopy. Several of the ISVs underwent regression at 30 hpf, starting from the junction at DA and extending dorsally towards DLAV (Fig. 3B). Tspan18 thus appears to regulate the stability of newly forming vessels during angiogenesis. A similar ISV instability phenotype has been noted in fish deficient in Notch-regulated ankyrin repeat protein (Nrarp) (Phng and Gerhardt, 2009).

**Fig. 4.**
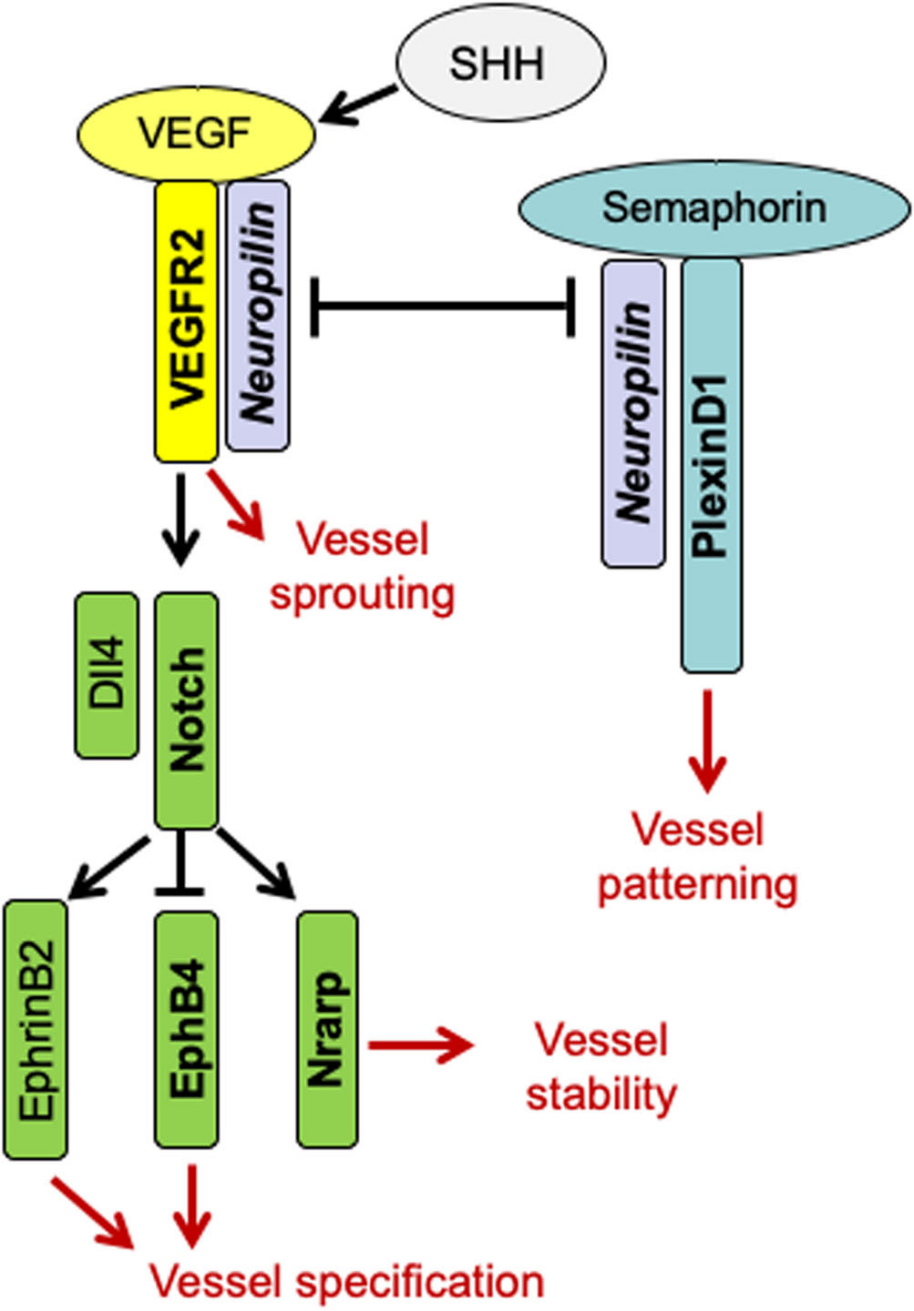
**A cartoon illustrating pathways involved in zebrafish embryonic angiogenesis.** SHH is a master regulator of angiogenesis and it regulates VEGF/VEGFR2. Neuropilin as a co-receptor is important for VEGF-VEGFR2 interaction and signaling, and regulates Semaphorin-PlexinD1 signaling. VEGFR2 signaling leads to vessel sprouting, whereas PlexinD1 signaling restricts angiogenesis and regulates vessel patterning. VEGFR2 induces regulates Dll4/Notch signaling, which in turn determines vessel specification (EphB4 on vein and EphrinB2 on artery) and vessel stability through Nrarp.

**Fig. 5.**
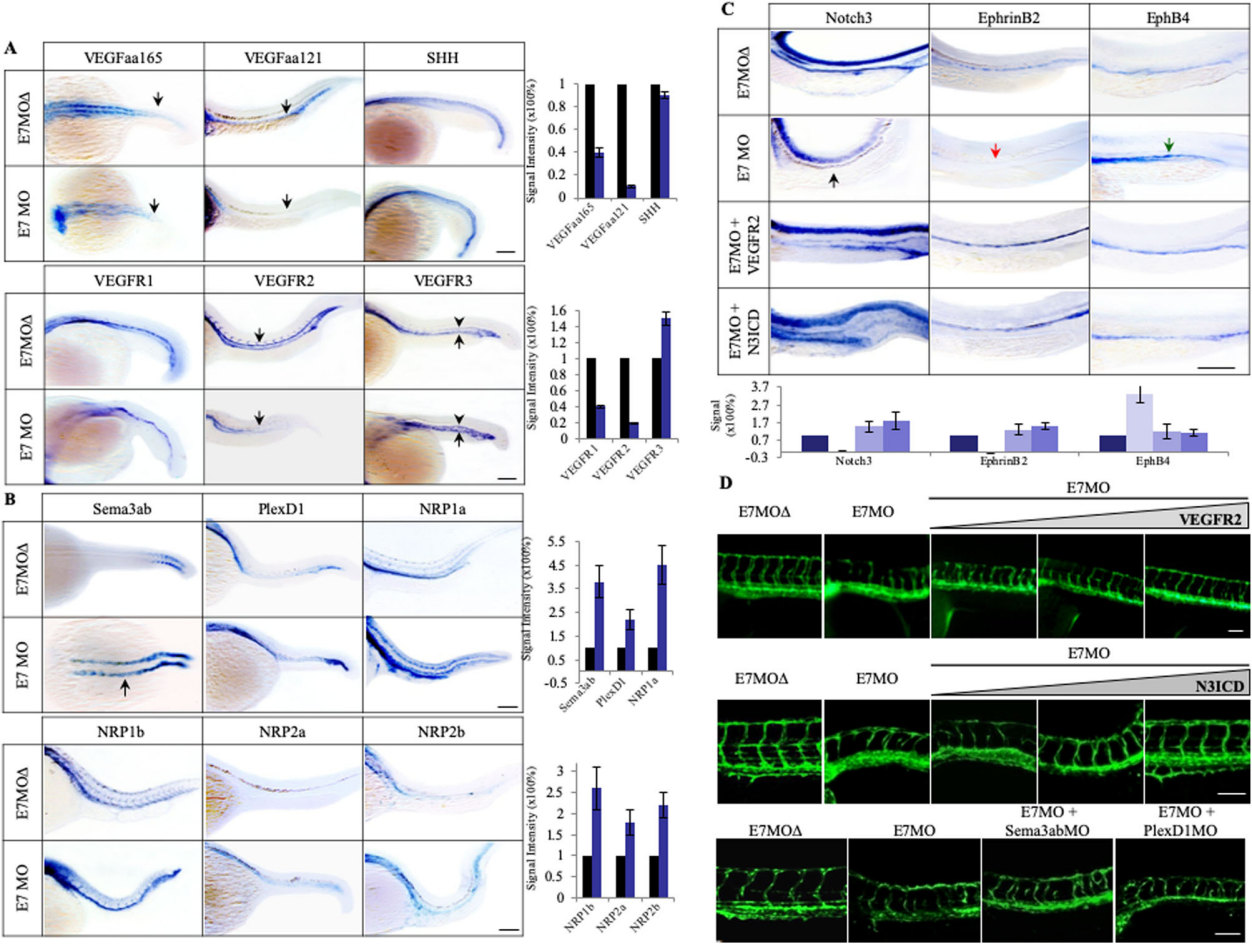
**Tspan18 functions through VEGF/VEGFR and Notch signaling pathways.** (A) Whole-mount ISH analysis of SHH, VEGFs and VEGFRs at 27 hpf in E7MO injected zebrafish. The expression of VEGFs especially VEGFaa121 and VEGFR2 was decreased (indicated by arrows) while the expression of VEGFR3 was increased in PCV (arrows) and ectopically expressed in DA (arrowheads). No significant changes were observed in SHH and VEGFR1 expression. Quantification of each genes is shown on the right (*n*>10). (B) Whole-mount ISH analysis of Sema3ab, PlexD1 and four Nrp isoforms in E7MO injected fish at 27 hpf. Sema3ab is highly induced in anterior somites (arrow). Quantification of each genes is shown on the right (*n*>10). (C) Whole-mount ISH analysis of Notch3, EphrinB2, and EphB4 at 27 hpf. In Tspan18 deficient fish, Notch3 (black arrow) and EphrinB2 (red arrow) were lost in the DA, while EphB4 was ectopically expressed in the DA (green arrow). These defects were rescued by co-injection of zebrafish VEGFR2 sense capped mRNA or Notch3 intracellular domain (N3ICD) mRNA. Quantification of each gene is shown at the bottom (*n*>10). (D) VEGFR2 (top) and N3ICD (middle) mRNA rescued E7MO-morphants in a dose-dependent manner (1.0, 1.5 and 2.0 ng per injection), whereas MOs targeting Sema3ab and PlexD1 (bottom) only partially rescued the defects (6 and 10 ng per injection respectively). Images of 27 hpf zebrafish are shown. Similar results were obtained from three independent experiments. All larvae lateral view. Anterior is to the left. Scale bars: 100 μm.

In order to investigate the mechanism of Tspan18 function, we examined the effect of Tspan18 deficiency on the key pathways involved in vascular development, including sonic hedgehog (SHH), VEGF/VEGFR, Semaphorin/Neuropilin/PlexinD1 and Notch (Fig. 4). These pathways regulate specific events in vessel sprouting, patterning, specification and stability. Quantitative RT-PCR and whole-mount ISH studies in E7MO embryos showed a marked decrease in VEGFaa165, VEGFaa121 (orthologs of human VEGFA), and VEGFR2 expression (Fig. 5A and Fig. S4). However, there was no change in the expression of SHH (Fig. 5A), a pathway upstream of VEGF (Lawson et al., 2002). The expression of VEGFR1 did not change in Tspan18-E7MO morphants, while VEGFR3, which is normally restricted to the venous endothelium by 24 hpf (Thompson et al., 1998), showed increased expression in PCV and ectopic expression in the DA (Fig. 5A). Tspan18 thus regulates VEGF/VEGFR pathway below SHH, directly or through Semaphorin–Neuropilin pathway.

Semaphorins bind Plexin and Neuropilin receptors, and regulate vessel patterning and cardiovascular development (Gelfand et al., 2009; Gitler et al., 2004). The Semaphorins can modulate VEGF signaling since Neuropilin receptors (Nrp1a, 1b and Nrp2a, 2b) are also the co-receptors for VEGFR2. Consequently, Semaphorins function as a sink for Nrp1, block VEGF binding and thus attenuate the VEGF signal. We therefore knocked down Semaphorin 3ab (Sema3ab) or PlexinD1 (PlexD1), the only Plexin receptor expressed in the endothelial cells (Torres-Vázquez et al., 2004; Gitler et al., 2004). We observed similar vessel-patterning defects and ectopic ISV branching with both sets of morpholinos (Torres-Vázquez et al., 2004). The phenotype is also observed in Tspan18 morphants. In Tspan18-deficient fish, quantitative RT-PCR (Fig. S4) and whole-mount ISH (Fig. 5B) showed an increase in the expression of Neuropilins, Sema3ab, and PlexD1. Interestingly, in Tspan18 morphants, Sema3ab, whose expression is normally restricted to the newly forming posterior somites at 20 hpf, not only had elevated expression in the posterior somites, but also had elevated and persistent expression in the anterior somites (Fig. 5B, arrow). Induction of Neuropilins, Sema3ab and PlexD1 in Tspan18-deficient embryos may contribute to diminution of VEGF pathway.

The upregulated expression of VEGFR3 in the arteries of Tspan18-deficient fish prompted us to investigate the role of Tspan18 in vessel specification. Arterial–venous specification is prominently regulated by the Notch pathway. Notch receptors and the ligand Dll4 are expressed in the arterial endothelium, and inhibition of the Notch signaling results in ectopic expression of venous markers (e.g. EphB4 and VEGFR3) and loss of arterial markers (e.g. EphrinB2) in the arteries (Roca and Adams, 2007). Impaired Notch signaling does not alter the formation of axial vessels similar to Tspan18-deficient fish (Siekmann and Lawson, 2007). We thus analyzed the levels of Notch3, which is an artery-specific marker in zebrafish (Lawson et al., 2001), Dll4 and downstream targets including Nrarp, EphrinB2 and its cognate receptor EphB4 at 27 hpf. The expression of Notch3, Dll4 and Nrarp was markedly decreased in E7MO embryos (Fig. 5C and Fig. S4). In addition, the expression of the arterial marker EphrinB2 was lost, and there was a reciprocal increase in the expression of the venous marker EphB4 (Fig. 5C and Fig. S4), similar to changes in gene expression observed after Notch inhibition (Lawson et al., 2001). Thus, Tspan18 may regulate the Notch pathway directly or secondarily to VEGF pathway modulation.

We next sought to determine if any of the pathways described above are responsible for the vascular phenotype observed in Tspan18-deficient zebrafish. First, E7MO was co-injected with the sense capped VEGFR2 mRNA at the 1–4-cell stage. Whereas injection of E7MO alone produced defects in 95% of ISV, co-injection of E7MO with VEGFR2 sense mRNA at 1.0, 1.5 and 2.0 ng/fish reduced ISV defects to 33%, 20% and 5% of injected animals, respectively (Table 1, Fig. 5D). The altered expression of artery- and vein-specific markers (EphrinB2 and EphB4) was also corrected by VEGFR2 mRNA (Fig. 5C). Co-injection of E7MO with zebrafish VEGFaa sense mRNA at 1.5 ng/fish in 215 embryos however did not revert the Tspan18 phenotype (Table 1) indicating that although VEGFR2 signaling is essential for Tspan18 function, VEGF alone is not sufficient to overcome the defects in the context of VEGFR2 deficiency. These data indicate that Tspan18 functions through modulation of VEGFRs or both but not VEGF alone.

**Table 1.**
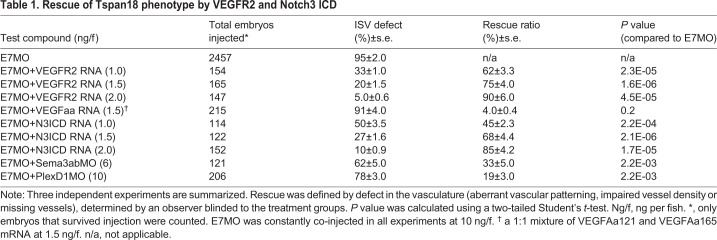
**Rescue of Tspan18 phenotype by VEGFR2 and Notch3 ICD**

We next introduced an activated form of Notch3 along with Tspan18 E7MO at the 1-4 cell stage to determine if Notch would rescue the vascular defects. A near complete rescue of vascular phenotype was observed with 2.0 ng/fish of Notch 3 intracellular domain (N3ICD) mRNA (Table 1 and Fig. 5D, middle), along with the normalization of artery and vein specific marker expression (Fig. 5C), supporting a critical role for the Notch pathway in mediating Tspan18 function.

To investigate whether the increase in Sema3ab signaling is responsible for the Tspan18-morphant phenotype, we injected morpholinos to Sema3ab or PlexD1 in Tspan18 morphants. The sequences and specificity of antisense MOs targeting Sema3ab, PlexinD1 have been described (Torres-Vázquez et al., 2004; Yu and Moens, 2005). Optimal concentrations for Sema3ab and PlexinD1 MOs were determined with serial titrations from 1 to 10 ng. Co-administration of E7MO with Sema3ab MO or PlexinD1 MO rescued only 33 and 19% of the animals, respectively (Table 1, Fig. 5D). Similarly, co-injection of NRP1a and NRP1b MOs only partially rescued E7MO phenotype (data not shown). These results indicate that Sema3ab/Nrp/PlexinD1 pathway is not likely to be the primary pathway mediating Tspan18 function.

**Fig. 6.**
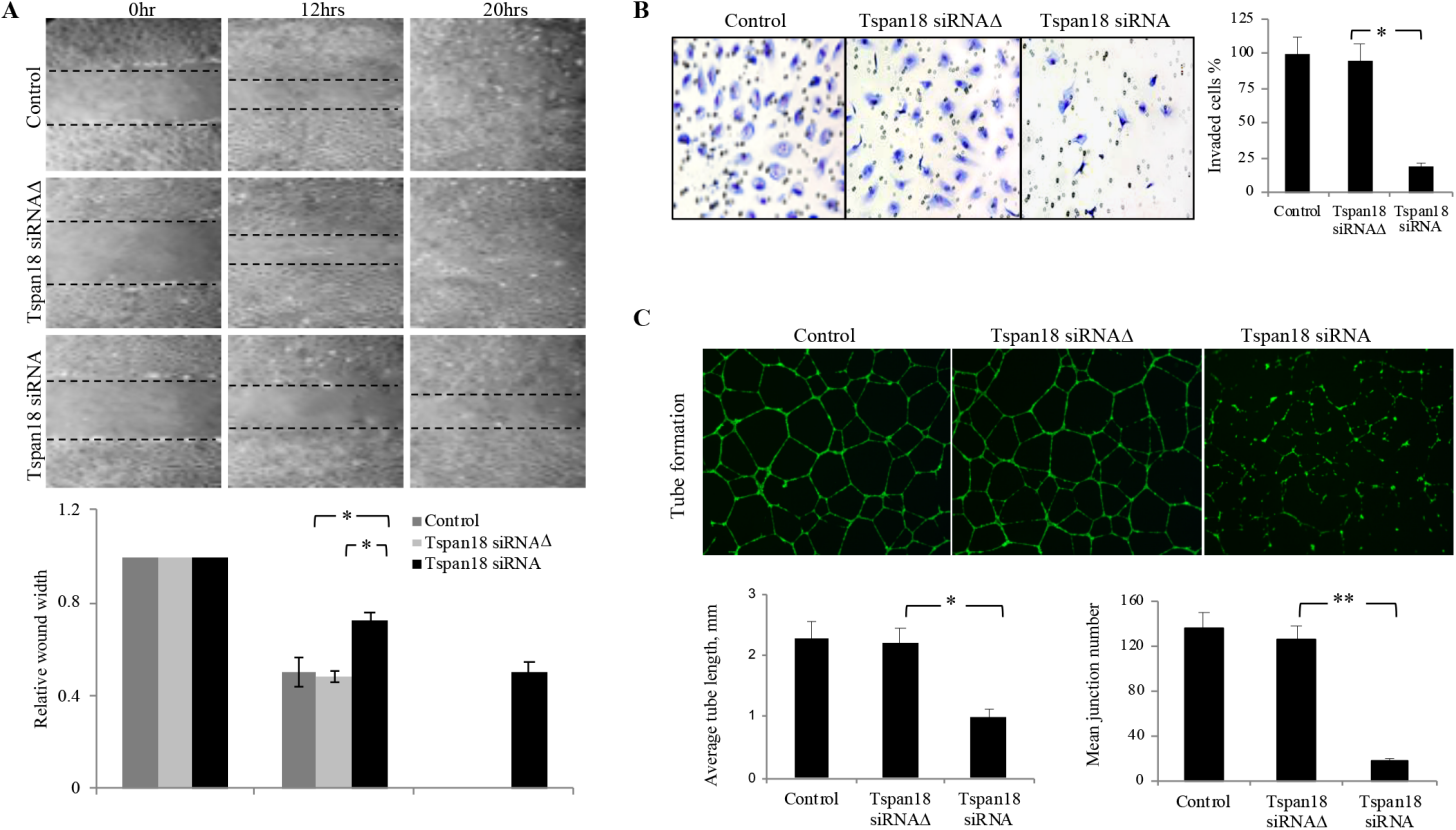
**Tspan18 knockdown inhibits angiogenesis *in vitro*.** (A) Tspan18 knockdown inhibited migration of HUAEC in an *in vitro* wound-healing assay. Transfection reagents served as a negative control. The margin of the wound is demarcated transwelled lines. Three independent experiments were performed and quantified wound width is shown (normalized to 0 h width). (B) Tspan18 knockdown inhibited invasion of HUAEC in transwell invasion assay. Quantification of invaded cells is shown on the right. (C) Tspan18 knockdown inhibited HUAEC tube formation on Matrigel. Quantification of tube length and number of junctions is shown on the bottom. The experiments in A–C were repeated three times with similar results. Error bars: s.d., *P<0.01, **P<0.002. *P* values were calculated using a two-tailed Student's *t*-test.

Having established the role of Tspan18 in Zebrafish *in vivo* in regulating arterial–venous specification, vessel patterning and stability, we investigated whether Tspan18 similarly regulates these pathways in human cells. We tested Tspan18 function *in vitro* in human endothelial cells for migration, invasion, and vascular network formation. Tspan18 siRNA or three-base mismatch control siRNA (Tspan18 siRNAΔ) was transfected into human umbilical arterial endothelial cells (HUAEC). A dose-dependent decrease in Tspan18 mRNA levels was observed after 48h transfection, with the maximal effect at 20 nM (Fig. S5A). The specificity of Tspan18 siRNA was also confirmed by immunoblot analysis in HEK293T cells stably expressing Tspan18 (Fig. S5B). In an *in vitro* wound-healing assay, Tspan18 siRNA markedly inhibited HUAEC migration (Fig. 6A). Tspan18 siRNA also resulted in a significant reduction (65%) in HUAEC invasion in a transwell invasion assay (Fig. 6B). Endothelial cells form tube-like structures when placed on matrigel, analogous to the formation of a vascular network *in vivo*. As shown in Fig. 6C, knockdown of Tspan18 markedly impaired the formation of the vascular network, as assessed by the number of branch points (junctions) and the lengths of tube-like structures.

**Fig. 7.**
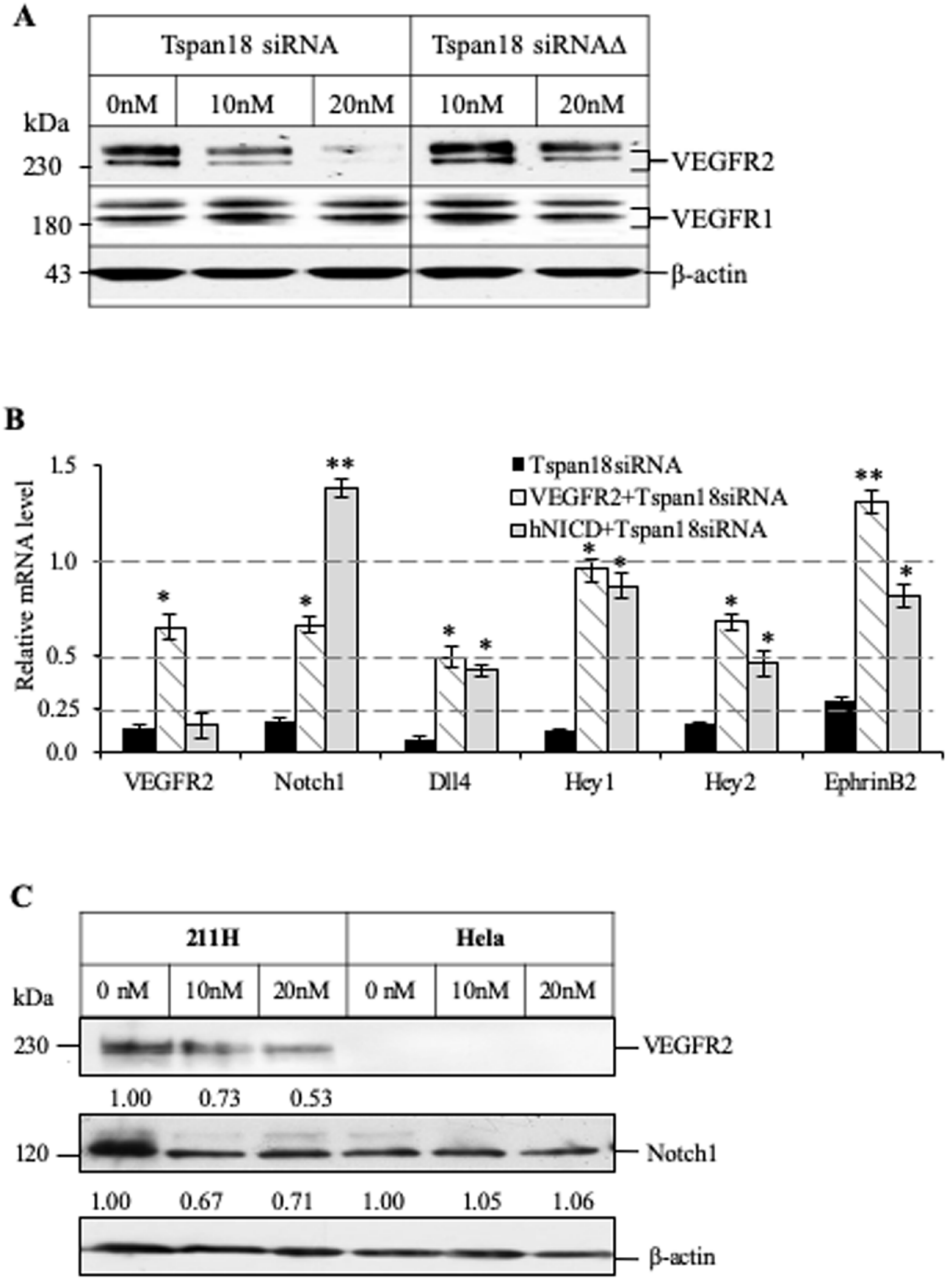
**Knockdown of Tspan18 by siRNA leads to downregulation of VEGFR2 and Notch1 *in vitro*.** (A) Western blot analysis of VEGFR2 and VEGFR1 expression in Tspan18 siRNA transfected HUAEC. A mutant siRNA (Tspan18 siRNAΔ) was used as siRNA control. β-actin served as loading control. (B) VEGFR2 and hNICD normalized the gene-expression changes in Tspan18-deficient HUAEC. VEGFR2 and hNICD were overexpressed in HUAEC transfected with Tspan18 siRNA. Gene expression was analyzed by quantitative RT-PCR, normalized to β-actin level and compared to that of Tspan18 siRNAΔ-treated HUAEC (set as 1.0). The experiments were repeated three times with similar results. Error bars: s.d., *P<0.01, **P<0.002. *P* values were calculated using a two-tailed Student's *t*-test. (C) Western blots showing the downregulation of Notch1 and VEGFR2 in VEGFR2-expressing mesothelioma cell line 211H by Tspan18 siRNA. No change in Notch1 is observed in cervical cancer Hela cell line that has no VEGFR2. This experiment was repeated three times with similar results. The immunoblots were quantified with Image J and the relative band intensity is shown.

We next examined the expression level of VEGF and Notch pathway genes in Tspan18-deficient endothelial cells. HUAEC cells treated with Tspan18 siRNA showed a dose-dependent reduction of VEGFR2 protein levels as compared to Tspan18 siRNAΔ treatment, while no change of VEGFR1 (Fig. 7A). Notch1, Dll4, EphrinB2, Hey1 and Hey2 (both Notch signaling effector genes) were also down-regulated in Tspan18 deficient HUAEC cells (Fig. 7B), suggesting the loss of arterial phenotype, a consequence of impaired Notch signaling. The expression of these artery-specific genes could be corrected by over-expression of VEGFR2 and activated Notch1 (intracellular domain of human Notch1, hNICD) (Fig. 7B), recapitulating the rescue by VEGFR2 and activated Notch3 over-expression in Tspan18-deficient zebrafish. Furthermore, to elicit the regulation of Notch signaling by Tspan18 is VEGFR2-dependent or not, we knocked down Tspan18 in VEGFR2-deficient cancer cell line Hela and didn't observe significant changes in Notch1, Dll4, EphrinB2 and EphB4 levels (Fig. 7C, Fig. S5C, and data not shown), whereas Notch1 was downregulated when knocking down Tspan18 in VEGFR2-positive cancer cell line 211H (Fig. 7C and Fig. S5C). These cancer cell lines may have contextual mismatch in understanding if Tspan18 targets VEGF or Notch pathway or both using endothelial cells deficient in VEGFRs or Notch receptors.

Angiogenesis is a critical component of wound healing. The process of angiogenesis is closely related to the formation of granulation tissue, which happens around 3 days post-wounding in mouse cutaneous wound repair, then angiogenesis has to cease when wound defect is filled with granulation tissue. To determine whether Tspan18 has a pathologic implication in the wound-healing process we tested Tspan18 mRNA and protein expression. Real-time RT-PCR demonstrated that Tspan18 was upregulated 1 day after injury; reached the peak at day 3; and fell down to basal level at day 7 (Fig. S6A). Consistent with mRNA expression, the elevated Tspan18 protein signals (Fig. S6B, upper, red color) right next to the wound area (lower, indicated by dot lines) were found around day 3 and fell down to basal level at day 7 post-wounding. The CD31 co-localization (upper panel, insert boxes) further confirmed the vessel structure. The fact that CD31 is expressed in both the existing vessels and newly formed capillaries, whereas Tspan18 is only expressed in newly formed capillaries, may explain the partially overlapping signals between Tspan18 and CD31. The temporal and spatial expression pattern of Tspan18 strongly suggests that Tspan18 plays a functional role in angiogenesis of wounding healing.

## DISCUSSION

We have determined that Tspan18 is prominently expressed in developing embryo vessels as well as adult wound healing. It regulates vessel patterning and arterial–venous specification, and is required for stabilizing newly forming vessels as judged by the regression of ISVs in Tspan18-deficient zebrafish. Reduction of Tspan18 in the endothelial cell results in the downregulation of VEGFR2, but defects caused by Tspan18 deficiency can be completely rescued by VEGFR2 overexpression, indicating that Tspan18 regulates the VEGF pathway, either directly or by modulation of Notch or related pathways. However, Tspan18 deficiency does not affect axial vessel development, which may be explained by incomplete inhibition of VEGF/VEGFR2 pathway. It is known that partial loss of VEGFA or VEGFR2 does not fully inhibit the establishment of axial vasculature, even though the sprouting angiogenesis is impaired (Carmeliet and Jain, 2000; Habeck et al., 2002).

Tspan18-deficient zebrafish have decreased expression of arterial markers such as EphrinB2 and Dll4 and unexpected induction of venous markers such as VEGFR3 and EphB4 in the arteries. These features are reminiscent of a switch from an arterial to a venous phenotype when the Notch pathway is inhibited. It is also known that loss of the Notch target gene Nrarp causes vessel regression (Phng and Gerhardt, 2009). In fact, Tspan18 knockdown results in reduction of Notch receptor, Notch ligands and downstream regulated genes including Nrarp. Furthermore, forced expression of Notch signaling through NICD rescues all of the vascular defects and arterial–venous specification, indicating a critical role of Notch signaling in the vascular function of Tspan18. The regulation of the Notch pathway by Tspan18 is likely through the VEGF pathway, based on the findings that VEGFR2 overexpression rescued both Notch expression and vessel defects, and Notch1 was not affected when Tspan18 was knocked down in VEGFR2-deficient cell lines. Tspan18 also regulates Sema3ab and its receptor PlexinD1 and Nrp1. However, downregulation of either Sema3a or its receptors produced only a partial rescue. The contribution of the Semaphorin pathway, if any, to Tspan18 function remains to be determined. In summary, we have identified Tspan18 as a novel gene expressed in developing vasculature, it regulates vessel specification, branching and stability.

## MATERIALS AND METHODS

### Animals

Tϋebingen wild-type zebrafish strain and Tg *fli1a:eGFP^y1^* transgenic fish were maintained as described previously (Lawson and Weinstein, 2002b). Embryos were staged as reported previously (Kimmel et al., 1995).

### Cell lines, constructs and transfections

Human umbilical artery endothelial cells (HUAECs) and human umbilical vein endothelial cells (HUVECs) were purchased from Lonza Walkersville, MD, USA, and maintained in corresponding medium provided by Lonza using University of Southern California Institutional Review Board approved tissue procurement protocol. HEK293T, H211, A2780, HOC7, MCF7, MDA-MB231, HT29, SCC15, Colo205, Hela and 211H cell lines were from ATCC. Human Tspan18 tagged with Myc was cloned into pcDNA3.1 (Invitrogen, Carlsbad, CA, USA). Mouse VEGFR2 in pCMV-SPORT6 was from Open Biosystems (Huntsville, AL, USA, Cat# MMM1013-65680). Transfection of HEK293T cells was performed with BioT (Bioland Scientific, Cerritos, CA, USA) according to the manufacturer's protocol.

### Whole-mount and ISH

ISH was performed as described previously with modifications (Drummond et al., 1998). Specifically, section *in situ* was performed using ISH kit (Biochain, Hayward, CA, USA) according to the manufacturer's protocol. DIG-labeled antisense and sense probes were synthesized by *in vitro* transcription using T7 and T3 RNA polymerase (Promega, San Luis Obispo, CA, USA). A 0.7 kb PCR fragment was amplified from the plasmid (BC076426) containing the full-length cDNA of Danio rerio Tspan18 and subcloned into pCR4.0 TOPO vector (Invitrogen, Carlsbad, CA, USA). Zebrafish antisense probes for VEGFaa, VEGFab, VEGFR2, VEGFR3, Notch3, EphrinB2a and EphB4a were kindly provided by Dr. Weinstein BM. Probes for Dll4 (Leslie et al., 2007), Sema3ab and PlexD1 (Torres-Vázquez et al., 2004) were synthesized as previously described. Images were obtained using an Olympus BX51 microscope equipped with a QImaging Retiga 2000R camera.

### MO

MOs (Gene Tools, Philomath, OR, USA) were designed to either block the translation initiation (ATGMO) or target exon 7 splice-donor site causing splicing defects of the mRNA (E7MO). The sequences of MOs are: 5′-AGCTCAGACAGTCCCCCTCCATGCT-3′ (ATGMO), 5′-AcCTCAcACAcTCCCCgTCCATcCT-3′ (five-base mis-match for ATGMO, ATGMOΔ), 5′-TGTAGAGTCATTGCTGACCTTGTTC-3′ (E7MO), 5′-TGTAcAGTg ATTcCTGAgCTTcTTC-3′ (five-base mis-match for E7MO, E7MOΔ). The primers spanning exon 7 (5′-TGGGCTGCTGTGGAGCCATTCG-3′ and 5′-CCACGATTGCA AGTGCTCCAGCC-3′) were used to verify the efficiency of E7MO. Each embryo was injected with 10 ng of the Tspan18 MOs.

### Tspan18 morpholino rescue experiments

Human Tspan18 full-length cDNA (BC019342.2), zebrafish VEGFR2 full-length cDNA (AY056466.1), zebrafish Notch3 ICD cDNA (NM_131549.2, nt.5236-7314) and the full-length cDNA of VEGFaa165 and VEGFaa121, kindly provided by Dr Ruowen Ge (Liang et al., 2001) were used for rescue experiments. Synthesis of all capped sense RNAs was made with the mMESSAGE mMACHINE kit (Ambion, Austin, TX, USA). The zebrafish Sema3ab (Sema3abMO) and PlexinD1 (Plxn^2215-2304^) morpholinos were synthesized as previously described. The activity of these morpholinos was documented by observing similar phenotypes as previously described (Torres-Vázquez et al., 2004; Yu and Moens, 2005). The safe dose (lacking non-specific toxicity) of each mRNA (VEGFR2 mRNA, N3ICD mRNA or a 1:1 mixture of VEGFaa121 and VEGFaa165 mRNA) and each MO (Sema3abMO, or PlexinD1MO) was predetermined, and subsequently mixed with E7MO or ATGMO prior to injection. The doses of E7MO and ATGMO were constant in all rescue experiments. Rescue was defined by the lack of vascular defects determined by an observer blinded to the treatment groups. All rescue experiments were repeated at least three times.

### Time lapse and confocal microscopy

Live embryos were mounted in 0.2% agarose supplemented with 0.1% tricaine on glass slides and imaged with a 20× objective on a Zeiss LSM 510 confocal microscope. Temperature of the chamber was adjusted to 28.5°C. Three-dimensional projections of confocal stacks were assembled using Zeiss LSM Image Browser.

### Cell and Tissue immunoblotting

Cells were harvested 48 h post-transfection and lysed with 1% CHAPS lysis buffer (10 mM Tris-HCl, pH 7.4, 150 mM NaCl, 1% CHAPS, 0.5 mM CaCl_2_, 0.5 mM MgCl_2_) supplemented with protease inhibitors cocktail (Pierce, Rockford, IL, USA). Tissues were lysed with modified RIPA buffer (10 mM Tris, pH 7.4, 100 mM NaCl, 1 mM EDTA, 1 mM EGTA, 1 mM NaF, 20 mM Na_4_P_2_O_7_, 2 mM Na_3_VO_4_, 1% Triton X-100, 10% glycerol, 0.1% SDS, 0.5% deoxycholate). Lysates were run on 4–20% Tris-glycine gradient gel (Bio-Rad), transferred onto nitrocellulose membrane (Bio-Rad) and probed with primary antibodies and horseradish peroxidase conjugated secondary antibodies (Rockland). An antibody against VEGFR1 (Cat# sc-271789) was purchased from Santa Cruz Biotechnology. Antibodies against VEGFR2 (Cat# 9698) and Notch1 (Cat#3608) were from Cell Signaling Technology. β-actin antibody was from Sigma-Aldrich (Cat# A5441).

### QRT-PCR

Total RNA was extracted using RNeasy mini kit (Qiagen, Valencia, CA, USA) from zebrafish embryos or cultured cells. First-strand cDNA was synthesized from 2 µg of total RNA with the kit from Fermentas and quantitative PCR was performed on the MX3000P real-time PCR system (Stratagene, La Jolla, CA, USA) using Brilliant II SYBR Green QPCR Mastermix (Stratagene, La Jolla, CA, USA) according to the manufacturer's instructions. All reactions were performed in triplicate. The amplification signal was normalized to β-actin. Primer sequences of studied genes are shown in the Supplemental Table.

### *In vitro* Tspan18 siRNA knockdown experiments

Human Tspan18 siRNAs were synthesized from Qiagen (Valencia, CA, USA). The sequences of siRNA are: 5′-CACGGTGATCCTCAACACCTT-3′ (Tspan18 siRNA) and 5′-CAt GGcGATaCTCAACACCTT-3′ (Tspan18 siRNAΔ). Transfection was performed with HiPerfect reagent (Qiagen) following the manufacturer's protocol.

### Migration assay

Tspan18 siRNA (20 nM) or Tspan18 siRNAΔ (20 nM) were transfected to HUAEC cells in 12-well plates. After 48 h cells were wounded by scraping a clear zone with a sterile pipette tip as previously described (Masood et al., 2006). The healing process was examined dynamically and recorded.

### Invasion assay

HUAEC cells were transfected with Tspan18 siRNA or Tspan18 siRNAΔ, and 6 h later 0.5×10^5^ cells were transferred into 8 μm Matrigel-precoated inserts with VEGF and bFGF as chemo-attractants. Following 6 h of incubation, the cells on the lower surface of the membrane were fixed and stained with Diff-Quick (DADE BEHRING, Cat# B4132-1A). The cells were counted in ten individual high-powered fields for each membrane under a light microscope as previously described (Liu et al., 2010).

### Endothelial cell tubular-formation assay

HUAEC cells transfected with Tspan18 siRNA or Tspan18 siRNAΔ for 48 h were seeded in triplicates on a 24-well plate (1×10^5^ cells/well) coated with matrigel (BD). After 6 h or 24 h incubation, cells were labeled with BD Calcein AM fluorescent dye. Images were obtained with an Olympus AX70 fluorescence microscope and Spot, Version 2.2.2 digital imaging system. Lengths of tubes and numbers of junctions were measured with ImageJ 1.42q (NIH, USA) software.

### Cutaneous wound healing

The back-skin hair of adult 25­­–30 g C57BL/6 mice was shaved and skin was sterilized with 70% ethanol. A single transverse full-thickness incision, 1 cm in length in each side of back was made by scalpel. The normal skin was collected for day 0 sample. One biopsy comprising the total wound area was collected from each animal on days 1, 2, 3, 5 and 7 post-wounding. The samples were bisected in the center, half prepared for frozen sections and half prepared for quantitative RT-PCR. All procedures were approved by Institutional Animal Care and Use Committee and performed in accordance with the Animal Welfare Act regulations.

## Supplementary Material

Supplementary information
