# Supplementary material for: Tetraspanin18 regulates angiogenesis through VEGFR2 and Notch pathways
Source: Biol Open. 2021 Feb 25;10(2):bio050096. doi: 10.1242/bio.050096 (PMC7928229; doi:10.1242/bio.050096)
Supplement: Supplementary information [file biolopen-10-050096-s1.pdf]

```
Mouse      MEGDCLSCMKYLMFVFNFFVFLGGACLLGVGIWVLVDPTGFREIVATNPLLTTGAYIVLA 60
Human      MEGDCLSCMKYLMFVFNFFIFLGGACLLAIGIWVMVDPTGFREIVAANPLLLTGAYILLA 60
Chick      MEGDCLSCMKYLMFLFNFFIFLGGACLLGVGIWVIVDPTGFREIVAANPLLFTGAYIMLA 60
Zebrafish  MEGDCLSCIKYLMFIFNFFIFLGGSFLLGVGIWVLVDPTGFREIVAANSLLFTGVYAILI 60

Mouse      MGGLLFLLGFLGCCGAVRENRCLLLFFFLFILIIFLVELSAAILAFIFREHLTREFFTKE 120
Human      MGGLLFLLGFLGCCGAVRENKCLLLFFFLFILIIFLAELSAAILAFIFRENLTREFFTKE 120
Chick      MGAMLFLLGFLGCCGAIRENKCLLLFFFMFILLIFLAELSAAILAFIFRENLTREFFTKE 120
Zebrafish  MGGMLFLLGFLGCCGAIRENKCLLLFFFMLILVIFLAELAVAILAFIFREHLTRDYFTKE 120

Mouse      LTKHYQGDNDTDVFSATWNSVMITFGCCGVNGPEDFKLASVFRLLTLDTEEVPKACCRRE 180
Human      LTKHYQGNNDTDVFSATWNSVMITFGCCGVNGPEDFKFASVFRLLTLDSEEVPEACCRRE 180
Chick      LKKHYVRNNDTHVFSSTWNSVMITFACCGVNGPEDFEAVPPLSHLPLE-ETTPEACCQRN 179
Zebrafish  LKTHYQGTNSTDVFTSTWNAIMTTFNCCGVNSAEDFDDQSLFRRLNPS-RIVPEVCCQR- 178

Mouse      PQTRDGVVLSREECQLGRNPFINKQGCYTVILNTFETYVYLAGAFAIGVLAIE------- 233
Human      PQSRDGVLLSREECLLGRSLFLNKQGCYTVILNTFETYVYLAGALAIGVLAIEEERAHSV 240
Chick      VQSREGMFVNRKACLEGDERFQNRQGCYTVILNSFETYVYLAGALAIGVLAIE------- 232
Zebrafish  ----TDLMMSKEECLRGIMPIRNK-GCYSAVVDYFETYIYMAGALAIVVLTIE------- 226

Mouse      ------LFLMVFAMCLFR--GIQ---- 248
Human      SQATGILYQLPASPQAFQSPGTLGATA 267
Chick      ------LFAMIFAMCLFR--GIQ---- 247
Zebrafish  ------LFAMVFAMCLFR--GIQ---- 241
```

Fig. S1. Protein sequence alignment of vertebrate (human, mouse, chick, and zebrafish) Tspan18. Not conserved amino acids are underlined.

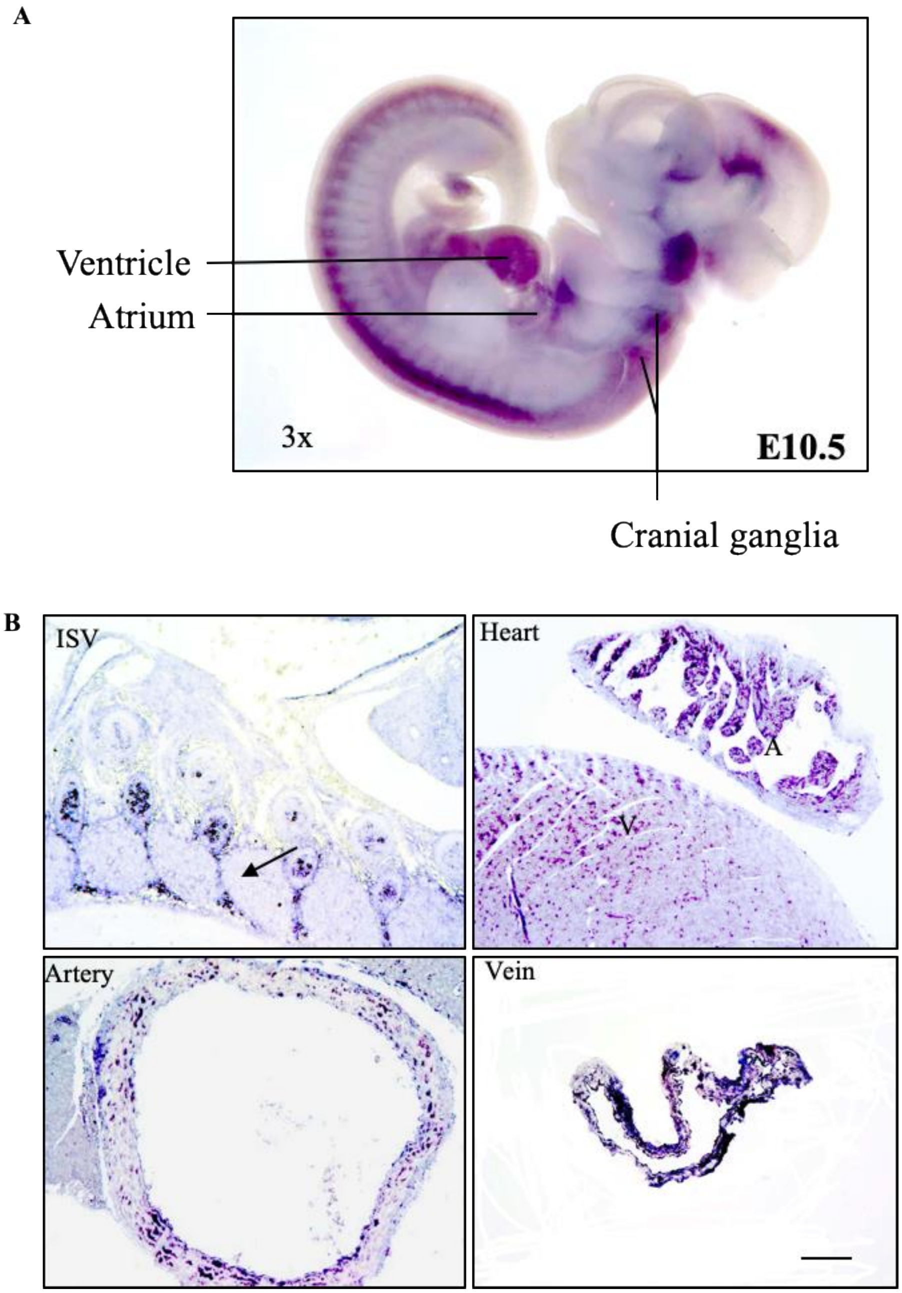

Fig. S2. In situ hybridization of mouse Tspan18 expression pattern. (A) Mouse whole embryo at age of E10.5. (B) Cross-section of mouse tissues. Expression was seen at intersomatic vessel (E13.5) and adult heart (V: ventricle; A: atrium), artery and vein.

Biology Open • Supplementary information

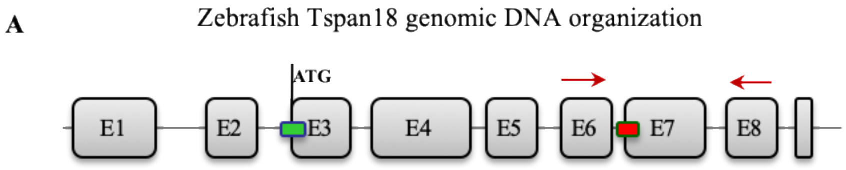

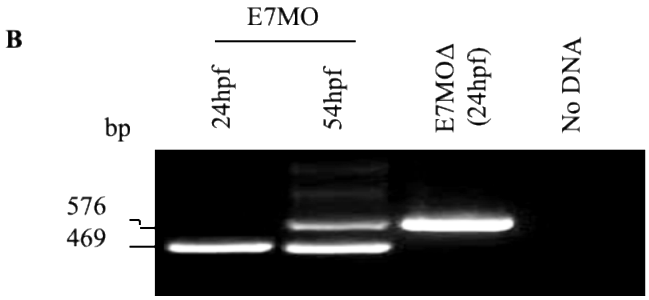

Fig. S3. Morpholino design for Tspan18 targeting in zebrafish. (A) Genomic DNA organization of zebrafish Tspan18 shows exons (gray rectangles) and introns (solid lines). Location of targeting sites of ATGMO (green rectangle) and E7MO (red rectangle), and primer set flanking exon 7 (red arrows) are shown. (B) E7MO treated embryos show a smaller PCR product (using primers shown in (A), indicating deletion of exon 7 compared to E7MOΔ.

Biology Open • Supplementary information

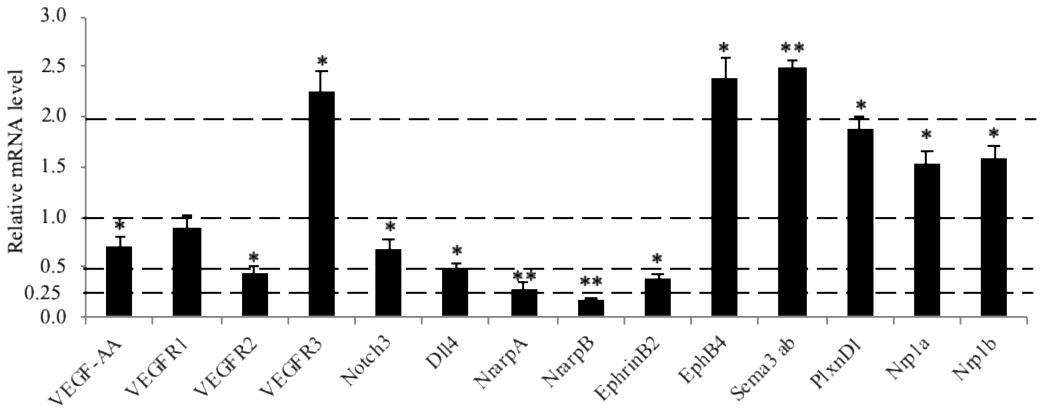

Fig. S4. Quantitative RT-PCR analysis of VEGF/Notch/Semaphorin pathway components of 27hpf zebrafish injected with E7MO or E7MO. Gene expression levels (N=5) were normalized to β-actin and compared to that of E7MOΔ injected fish (set as 1.0). Error bars represent SD. *P* value was calculated using two-tailed Student's *t*-test. *: *P*<0.02; **: *P*<0.005.

Biology Open • Supplementary information

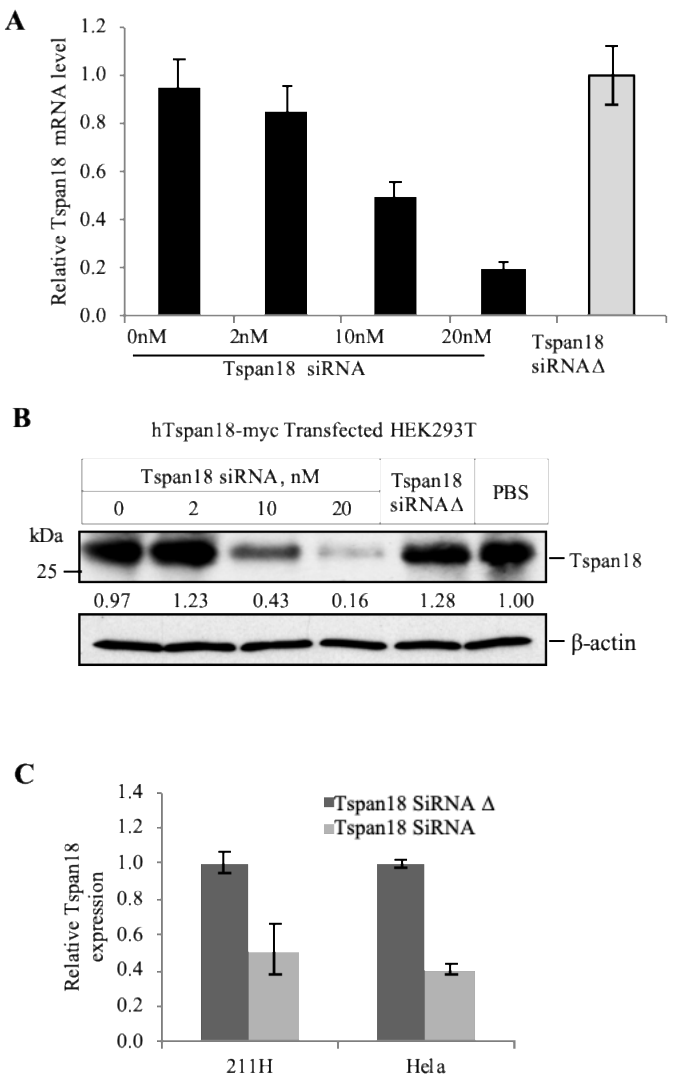

Fig. S5. Knockdown of Tspan18 by siRNA *in vitro*. (A) The dose-dependent knockdown of Tspan18 by siRNA was confirmed in HUAEC by quantitative RT-PCR. Tspan18 siRNAΔ (20 nM) and transfection reagents only (0 nM) served as controls. Expression level was normalized to β-actin. (B) The dose-dependent knockdown of Tspan18 by siRNA was confirmed in HEK293T cells stably expressing hTspan18-myc protein by western blot analysis. Tspan18 siRNAΔ (20 nM) and transfection reagent only (0 nM) served as negative controls. The quantification was performed with ImageJ (NIH) and normalized to PBS sample. (C) Tspan18 knockdown by siRNA (20 nM) was confirmed with quantitative PCR in 211H and Hela cell. Tspan18 siRNAΔ was used as a negative control.

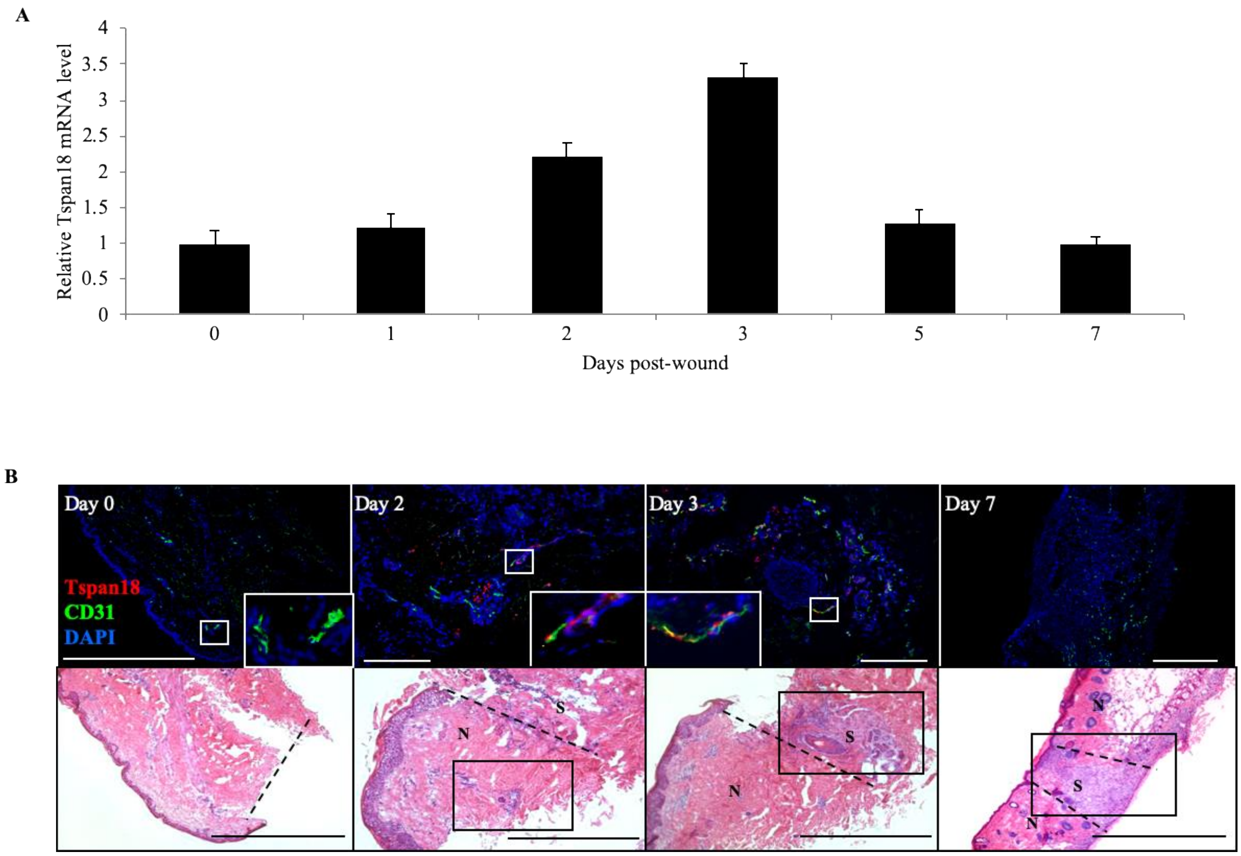

Fig. S6. Tspan18 is up-regulated in skin wound healing. (A) Quantitative RT-PCR of Tspan18 level during skin wound healing. (B) Immunofluorescent staining of Tspan18 (red), CD31 (green), and DAPI (blue) of skin wounds.

Biology Open • Supplementary information

Table S1. Real-time RT-PCR primer sequences

Gene	Forward Primer (5'-3')	Reverse Primer (5'-3')
hTspan18	TGGAGCACCATGGAAGGCGACTG	TGACCGAGTTCCAGGTGGCAGAG
hTspan12	ATCGCTGCCGTTTTGCCCTTGGG	GCAATCATGACCGGATGAACCAC
hVEGFa	ACCTCCACCATGCCAAGTGGTCC	TGCCCCTTTCCCTTTCCTCGAAC
hVEGFR1	TCGCGCTCACCATGGTCAGCTAC	CAGGGAATGACGAGCTCCCTTCC
hVEGFR2	ATGCAGAGCAAGGTGCTGCTGG	GACCGAGGCCAACTGAGTTTCC
hVEGFR3	CACGGAGGAGTCACACGTCATCG	TGCGAGCGCAGCGTGACATTGAG
hNotch1	GGTGAGACCTGCCTGAATG	GTTGGGGTCCTGGCATC
hNotch4	CAGCCCAGTGGGTATCTCTG	GTTGTGACAGGGTTGGGACT
hDll4	AGGCCTGTTTTGTGACCAAG	GTGCAGGTGTAGCTTCGCT
hHey1	TGGATCACCTGAAAATGCTG	CGAAATCCCAAACTCCGATA
hHey2	TTTGAAGATGCTTCAGGCAA	GGCACTCTCGGAATCCTATG
hEphrinB2	TCCGTGTGGAAGTACTGCTG	ACCAGTCCTTGTCCAGGTAGAA
hEphB4	ATGCCCGTCATGATTCTCAC	ACGAGCTGGATGACTGTGAA
hActb	GCACAGAGCCTCGCCTT	GTTGTCGACGACGAGCG
zTspan18	TAGGGGTGGGAATCTGGGTGCTG	AACTGGATTCCCCGAAACAAACAC
zVEGF-AA	AGAGTGCGTGCAAGACCCGAGAG	GGCCTGCATTCACACTTGGTGTG
zVEGFR1	GAAGGTGCAGTCGGAGATCCCAG	GCATCTCGCCCTGTAACGTGTGC
zVEGFR2	GAGCCTCGGGTCAATGCTGTTCC	TCTGAAGGTCTGGGGACCTGCTG
zVEGFR3	GGTGCTCAACTGCACTGCACTGG	GTCCCACTGGAAATCCACACCAG
zNotch3	TGGTTCGCTCTGTCAGCATCTGG	GACATTGTGTACGGGCACAGGGC
zDll4	ATGGCAGCTTGGCTCACCTTTCTC	CCTGCCATCCTTCTCTGCATACAC
zNrarpA	ATGAGCCAGGCGGATATATCGACG	TCACCGCGCGCCGGACGAGTACTTG
zNrarpB	ATGAGTCAGGCGGACATGACCTGC	TCAGAGCGCGCTTGATGAGTATTTC
zEphrinB2	ACTGCTCAACTGTGACAAGCCG	TGCGCCGAGTGCTTCTGATGAC
zEphB4	AGTGGACGACACACTCTCGCTC	AGACGGATAGTGAGCGCGTGAGC
zSema3ab	GAGATGAATGCAAGTGGGCCGGG	CCGGCCCATGAAGTCAGCTGATG
zPlxnD1	ACCTGTACCAGCTGAACGCGACG	GATCTCCGGGGTGTTCTCGAAGC
zNrp1a	GGAGTCTTCTGCCGATACGACCG	GAGAAATGGCTCCCTGTGTGCCG
zNrp1b	TGACATGGAGCCGGACACAACGG	TCAGTCTGGAGCGCAGTCGAGAC
zActb	ATGAGCTCCGTGTTGCCCCTGAG	AGTCCAGGGCCACATAGCACAGC

Biology Open • Supplementary information
